# Single vascular insult aggravates amyloid accumulation with age triggering glial activation in AD mice

**DOI:** 10.1002/alz.092235

**Published:** 2025-01-03

**Authors:** Latha Diwakar, Mayank Gupta, Ankita Talukdar, Rehab Hussain

**Affiliations:** ^1^ Centre for Brain Research, Indian Institute of Science, Bengaluru, Karnataka India; ^2^ Manipal Academy of Higher Education, Manipal, Karnataka India

## Abstract

**Background:**

Vascular pathology is often seen in cases of mixed dementia affecting elderly population including Alzheimer’s disease (AD). AD is generally characterized by the presence of amyloid‐β (Aβ) plaques and tau deposits. However, many factors influence the onset and progression of AD pathology. In the present study, we are looking for brain vascular pathology like blood brain barrier (BBB) breach by vascular insult and associated microglial activation in small vessels increase risk of developing pathology over age. It is often hypothesized that in presence of increased Aβ, vascular insults could synergistically trigger neuroinflammation leading to cognitive impairment accelerating neurodegeneration.

**Method:**

Learning and memory deficits were performed in slow progressing APPswe mice injected with 2µg/2μl of ET‐1(endothelin‐1) bilaterally into lateral ventricles after 30 days of ET‐1 injection. BBB structural changes were assessed by immunohistochemistry for markers of endothelial cells and pericytes. Aβ plaque load and vascular deposition of Aβ were tested to understand the effect of vascular insult on AD pathology in double transgenic J20 mice. Further, microglial activation was estimated using specific markers.

**Result:**

BBB breaching was increased after vasoconstriction following ET‐1 injection after 30 days with increasing age causing memory deficits in AD mice. The extent of structural changes in BBB increased with age indicating the susceptibility with mutation. Aβ plaque load and glial activation was increased in J20 mice after ET‐1 injection.

**Conclusion:**

APP mutation increases susceptibility to ET‐1 induced single vascular insult bringing about BBB leakage, memory deficits, increased Aβ aggregation and glial activation with increasing age given enough time of 30 days to rescue. However, there was rescue in case of wild type mice.

